# Paramagnetic Probing of Humic Acid Supramolecular Structure: Iron (III)–Induced Rearrangements Revealed by Solid‐State ^13^C NMR Spectroscopy

**DOI:** 10.1002/mrc.70011

**Published:** 2025-07-10

**Authors:** Pellegrino Conte, Calogero Librici

**Affiliations:** ^1^ Department of Agricultural, Food and Forest Sciences University of Palermo Palermo Italy

**Keywords:** CPMAS ^13^C NMR, cross polarization, humic conformation, humic substances, molecular dynamics, rotating‐frame relaxation time, supramolecular structure

## Abstract

Humic acids (HAs), key components of soil organic matter, undergo significant conformational rearrangements upon complexation with metal ions, yet the molecular‐scale dynamics of these interactions remain poorly understood. In this study, solid‐state 13C CPMAS NMR spectroscopy was employed to probe Fe (III)–induced structural changes in a soil‐derived HA, focusing on cross‐polarization time constants (*T*_*CH*_) and proton rotating‐frame relaxation times (*T*_₁*ρ*_[*H*]) as indicators of spatial and dynamic reorganization. Exponential decay analysis of *T*_*CH*_ versus Fe/C ratios revealed distinct binding hierarchies: carboxyl groups (187–163 ppm) exhibited the strongest response (decay constant *k*_*TCH*_ = 3.5), reflecting Fe (III)–driven local compaction and rigidification, whereas aromatic (163–92 ppm; *k*_*TCH*_ = 2.5) and oxygenated aliphatic (92–46 ppm; *k*_*TCH*_ = 1.5) domains showed intermediate sensitivity. Aliphatic carbons (46–0 ppm; *k*_*TCH*_ ≈ 0.05) remained inert, confirming their exclusion from metal coordination. Complementary *T*_1*ρ*_(*H*) data highlighted the role of Fe (III) in restricting molecular mobility, particularly in carboxyl and aromatic regions.

These findings support a model in which Fe (III) acts as a supramolecular cross‐linker, selectively rigidifying HA domains through primary carboxylate binding and secondary aromatic interactions, whereas aliphatic regions retain dynamic freedom. The study demonstrates that paramagnetic Fe (III) not only perturbs NMR detectability but also serves as a structural probe, with *T*_*CH*_ and *T*_1*ρ*_(*H*) providing quantitative metrics for metal‐induced reorganization. This work advances the mechanistic understanding of organo–mineral interactions in soils and highlights NMR relaxation parameters as powerful tools for characterizing environmental organic matter.

## Graphical abstract

Fe (III) complexation induces conformational compaction of humic acid by selectively rigidifying carboxyl and aromatic domains. Solid‐state ^13^C NMR relaxation (*T*_*CH*_ and *T*₁*ρ*[*H*]) reveals domain‐specific dynamics, supporting a supramolecular model, where Fe (III) acts as a molecular cross‐linker in natural organic matter.

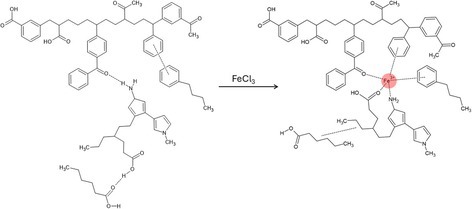

## 1 Introduction

Humic acids (HAs), major components of soil organic matter, are pseudo‐high molecular weight, polydisperse substances composed of aromatic and aliphatic domains, with a variety of functional groups such as carboxylic and phenolic moieties [1, 2, 3, 4, 5, 6]. Their structure, both in solution and in the solid state, is highly sensitive to environmental variables, including pH, ionic strength, and the presence of metal ions [6, 7]. Among these, metal–humic interactions are particularly significant, influencing metal speciation, bioavailability, and mobility in soils and natural waters [1, 8, 9, 10, 11, 12, 13].

The effect of metal complexation on the conformational organization of HAs has been extensively investigated [6, 14, 15, 16, 17]. It is now recognized that polyvalent cations—particularly transition metals such as Fe (III), Cu (II), and Al (III)—can act as cross‐linkers between distant functional groups, thereby inducing aggregation or compaction of the humic superstructure [16, 17, 18]. These structural rearrangements substantially affect the physicochemical properties of humic matter, including solubility, charge density, and interactions with pollutants or nutrients [6].

Recent studies have employed a range of spectroscopic and scattering techniques—such as dynamic light scattering (DLS), small‐angle X‐ray scattering (SAXS), electron paramagnetic resonance (EPR), and nuclear magnetic resonance (NMR)—to investigate these phenomena [19, 20, 21, 22, 23]. Paramagnetic metal ions like Fe (III) and Mn (II) offer dual advantages: They perturb the conformation of humic pseudo‐macromolecules and serve as NMR‐active probes of spatial organization, due to their magnetic susceptibility [24, 25, 26].

Traditional NMR relaxation experiments based on *T*_1_ and *T*_2_ provide valuable but spatially averaged information on molecular mobility. *T*_1_ relaxation is typically too slow and insensitive to segmental dynamics, whereas *T*_2_ measurements are affected by scalar J‐couplings, incomplete coherence refocusing, and MAS‐dependent artifacts [27, 28]. These limitations are particularly problematic when studying humic substances, which are not classical macromolecules but supramolecular associations of relatively small molecules stabilized by weak noncovalent interactions [1, 7, 29]. As a result, humic matter exhibits structurally heterogeneous and dynamically variable domains that challenge conventional relaxation‐based analysis [2].

A more suitable approach is provided by variable contact time (VCT) ^13^C CPMAS NMR experiments. This technique enables the construction of cross‐polarization build‐up curves that, through appropriate modeling, yield both the cross‐polarization time constant (*T*_*CH*_) and the longitudinal relaxation time in the rotating frame (*T*_1*ρ*_(*H*)) [30, 31, 32]. In particular, *T*_*CH*_ captures the efficiency of polarization transfer, which depends on proton–carbon proximity and local segmental rigidity. Conversely, *T*_1*ρ*_(*H*) responds to motional processes on the microsecond‐to‐millisecond timescale, revealing differences in dynamic flexibility across humic domains [23].

The robustness of this combined approach has been validated by key studies. For instance, Dria et al. [33] showed that ramped CP sequences combined with short contact times enhance both sensitivity and selectivity, enabling semiquantitative comparisons even in highly disordered organic matrices. Moreover, van Lagen and de Jager [34] demonstrated that the use of multiple contact times, together with extrapolation techniques and optimized steady‐state conditions, significantly improves the accuracy of CPMAS‐based quantification in soil‐derived humic substances.

Taken together, *T*_*CH*_ and *T*_1*ρ*_(*H*) offer a spectroscopically integrated and spatially resolved framework for probing HA domain structure and its response to metal complexation. This is especially valuable given the inherent microheterogeneity of humic matrices and their dynamic response to environmental perturbations [35, 36].

Within this framework, we investigated how Fe (III) complexation modulates the supramolecular organization of a well‐characterized soil‐derived HA. Using solid‐state ^13^C NMR spectroscopy and model‐based analysis of cross‐polarization dynamics, we examined the effect of increasing Fe/C ratios on the spatial distribution and dynamic behavior of carbon moieties, with the aim of elucidating the nanoscale structural reorganization induced by paramagnetic metal ions, a key process underlying the functional behavior of humic substances in natural environments.

## 2 Materials and Methods

### 2.1 Humic Acid Samples

A HA was extracted by standard alkaline extraction from a *Typic Fulvudand* soil sampled in the ancient volcanic area of Vico, near Rome (Italy). The soil characteristics are described elsewhere [37]. Extraction, purification, and elemental characterization of the HA are detailed in the Supporting Information.

The HA acidity, determined as reported in the Supporting Information, was used to prepare Fe‐humate complexes with Fe/C ratios of 0, 1.0, 1.5, 2.5, 3.2, 4.0, 5.0, and 7.5. Both the bulk HA and the Fe‐HA complexes were subsequently analyzed by CPMAS ^13^C NMR spectroscopy.

### 2.2 CPMAS ^13^C NMR Spectroscopy

Solid‐state ^13^C NMR spectra were acquired using a Bruker AMX 300 spectrometer (Bruker Analytische Messtechnik GmbH, Rheinstetten, Germany) equipped with a 7‐mm‐wide‐bore probe, operating at 75.478 MHz on carbon‐13. The samples were packed in 7‐mm zirconia rotors with Kel‐F caps and spun at 5000 ± 2 Hz. This spinning rate was selected as a practical upper limit for this instrumental configuration in order to ensure stable, symmetric rotation, and prevent potential probe damage due to rotor instability [38]. In addition, higher spinning speeds can induce vortex effects in soft, heterogeneous materials such as humic substances, leading to spectral artifacts and compromised data quality [23]. We are fully aware of the presence of spinning sidebands, which cannot be completely removed at the selected spinning speed. However, the use of the well‐established CP‐TOSS pulse sequence, commonly applied to suppress sidebands and enhance spectral clarity, was deliberately avoided. This is because the sequence also suppresses signal components that overlap with the sideband regions, potentially eliminating meaningful spectral information [39, 40]. Consequently, CP‐TOSS is more appropriate for qualitative assignments than for quantitative or dynamic analyses of supramolecular systems, as required in the present study.

A ^13^C spectral width (SW) of 125,000 Hz centered at 8240 Hz (O1), a time domain (TD) of 8 k over an acquisition time (AQ) of 35 ms, a recycle delay (D1) of 2 s, and a ^1^H 90° pulse of 3.5 s with an attenuation level (PL) of −1.5 db, as per standard Bruker calibration protocols for this probe configuration. The recycle delay of 2 s was decided according to the results from Berns and Conte [41]. Two‐thousand scans (NS) were used for spectra acquisition. An ^1^H Ramp CP pulse sequence was applied to account for the Hartmann–Hahn variations at the selected rotor spin rate. In particular, an ascending Ramp of 12 kHz was applied according to Berns and Conte [41]. The variable contact time (VCT) experiments were conducted across a contact time range of 0.025–6.0 ms. All the contact times (CT) were selected based on the relationship:

$${\mathrm{CT}}_{i}={\mathrm{CT}}_{\min}\times {\left(\frac{{\mathrm{CT}}_{\max}}{{\mathrm{CT}}_{\min}}\right)}^{\frac{i-1}{N-1}},$$

where ${\mathrm{CT}}_{\min}=0.025\;\mathrm{ms}$, ${\mathrm{CT}}_{\max}=6\;\mathrm{ms}$, i is an entire number ranging between 1 and N, and N=20. XWinNMR 2.0 software (Bruker Biospin, the Netherlands) was used for free induction decays (FIDs) acquisition, whereas MestRe‐C software (Version 4.9.9.9, Mestrelab Research, Santiago de Compostela, Spain) was run for data processing. All the FIDs were Fourier transformed by applying an exponential filter function with a line broadening (LB) of 50 Hz and an 8‐k zero filling. Spectra were baseline corrected with a first‐order polynomial with Bernstein algorithm [42].

Spectra were divided into four chemical shift regions based on literature assignments [30, 31, 32]: 187–163 ppm (carboxyl carbon, –COOH), 163–92 ppm (aromatic carbon, acetals, and lignin‐like systems), 92–46 ppm (N‐ and O‐alkyls), and 46–0 ppm (aliphatic carbon). Spinning side bands (SSB) in the regions 250–229 ppm (carboxyl SSB) and 229–187 ppm (aromatic SSB) were accounted for according to Conte et al. [30] only in the spectrum of the bulk HA. No SSBs were detected in Fe‐HA complexes due to lower spectral sensitivity.

### 2.3 Evaluation of the Cross Polarization (*T*_*CH*_) and the Proton Longitudinal Relaxation Time in the Rotating Frame (*T*_1*ρ*_[*H*])

The cross‐polarization time constants (*T*_*CH*_) and the proton rotating‐frame relaxation times (*T*_₁*ρ*_[*H*]) were determined by fitting signal intensities from the VCT experiments to the following equation [2, 28]:

(1) $$S\left(t\right)=S_{0}\alpha^{-1}\left(1-\exp\left(\alpha \frac{t}{T_{\mathrm{CH}}}\right)\right)\exp\left(-\frac{t}{T_{1\rho }\left(H\right)}\right),$$

where $S\left(t\right)$ is the signal area at contact time t, $S_{0}$ is the equilibrium magnetization, $T_{\mathrm{CH}}$ is the cross‐polarization time, and $T_{1\rho }\left(H\right)$ is the proton rotating‐frame relaxation time associated with each spectral region. Finally, $\alpha =1-\frac{T_{\mathrm{CH}}}{T_{1\rho }\left(H\right)}$. Fitting was performed using MestRe‐C (Version 4.9.9.9) data analysis tool, yielding correlation coefficients (*R* ^2^) greater than 0.95 for all fits.

### 2.4 *T*_*CH*_ and *T*₁ *ρ* (*H*) Trends as a Function of Fe/C Ratio

The $T_{\mathrm{CH}}$ and $T_{1\rho }\left(H\right)$ values obtained from Equation (1) were related to the corresponding Fe/C ratios. An exponential function of the form:

(2) $$y=y_{0}+A_{1}\exp\left(-k_{i}x\right),$$

was used to interpolate the data points. In Equation (2), y represents the $T_{\mathrm{CH}}$ or $T_{1\rho }\left(H\right)$ values for each chemical shift region, $y_{0}$ is the offset, $A_{1}$ is a pre‐exponential factor corresponding to the maximum value of $T_{\mathrm{CH}}$ or $T_{1\rho }\left(H\right)$, and *kᵢ* is the decay constant (*k*_*TCH*_ or *k*_*T*₁ *ρ* [*H*]_) describing the rate of decrease as a function of the Fe/C ratio reported in the *x* axis. All interpolations yielded *R* ^*2*^ values between 0.80 and 0.95.

## 3 Results and Discussion

### 3.1 Qualitative Evaluation of the CPMAS ^13^C NMR Spectra

**FIGURE 1:**
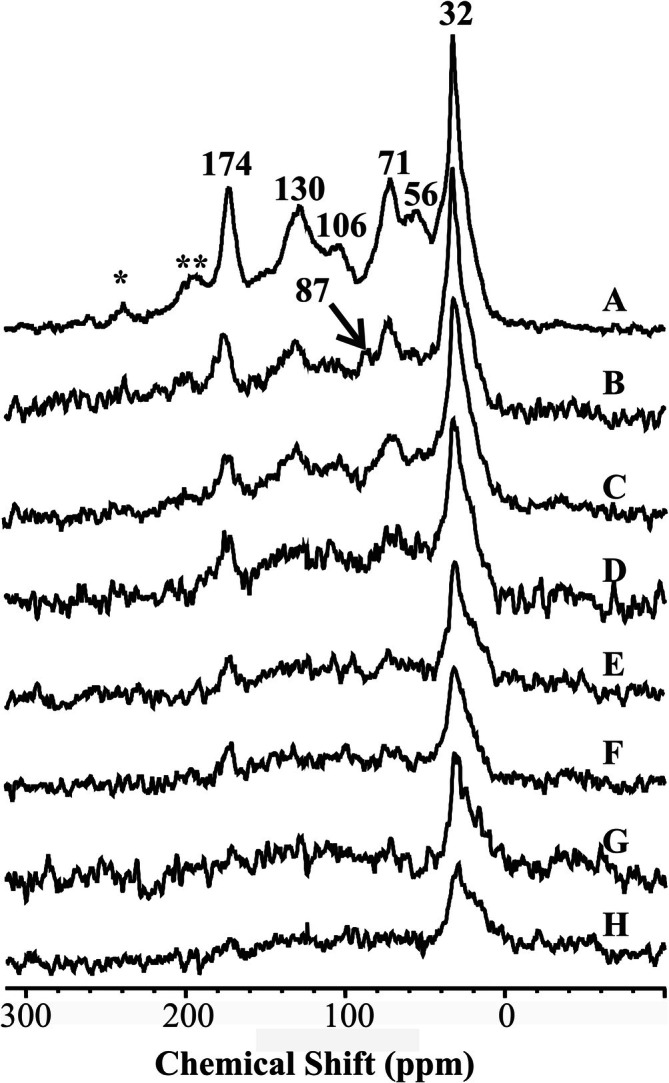
CPMAS ^13^C NMR spectra of the different Fe‐humic complexes. The spectra from (A) to (H) refer to the following Fe/C ratio: 0, 1, 1.5, 2.5, 3.2, 4.0, 5.0, and 7.5.

The solid‐state spectra in Figure 1 display the CPMAS ^13^C NMR profiles of the bulk HA (Figure 1A) and of its complexes with increasing amounts of paramagnetic Fe (III) (Figure 1B–H). As expected, a progressive reduction in spectral sensitivity is observed as the Fe/C ratio increases. This is a well‐documented consequence of the presence of paramagnetic species in proximity to NMR‐detectable nuclei, which induce line broadening and signal attenuation [43].

Two primary mechanisms have been proposed to explain this phenomenon [43]. The first involves perturbations in magnetic field homogeneity: Paramagnetic centers distort the local field, causing chemically equivalent nuclei to resonate at slightly different frequencies depending on their spatial location. This results in the merging of resonances into broad, unresolved bands with diminished intensity. The second mechanism is based on nuclear–electron spin interactions, which accelerate both longitudinal (*R*_1_) and transverse (*R*_2_) relaxation processes, thus shortening *T*_1_ and *T*_2_. Although reduced *T*_1_ values may facilitate faster acquisition by enhancing polarization transfer, shortened *T*_2_ times lead to significant broadening of signals and, in extreme cases, complete loss of detectable resonance.

These paramagnetic effects are particularly detrimental in solid‐state NMR, where chemical shift anisotropy and strong dipolar couplings—especially in heterogeneous materials like HAs—already challenge spectral resolution [23]. Even fast magic angle spinning (MAS) cannot fully compensate for the line broadening caused by unpaired electrons. In this context, the progressive degradation of spectral quality observed across the Fe‐HA series is fully consistent with expectations for systems containing increasing concentrations of Fe (III).

This behavior mirrors observations by Gonçalves et al. [44], who demonstrated that in iron‐rich *Ferralsols*, high paramagnetic content leads to substantial suppression of ^13^C NMR signals. Their work showed that paramagnetic iron oxides, when not removed by chemical treatment (e.g., HF extraction), interfere with magnetization transfer in cross‐polarization experiments by rapidly relaxing nearby protons. In such cases, if the ^1^H relaxation times become shorter than the CP contact time, efficient transfer to ^13^C is hindered, and signals are selectively quenched—particularly those of carbons spatially close to Fe centers. Although the Fe in our samples is intentionally added rather than inherent, a comparable effect is evident: Signal loss is most pronounced in spectral regions associated with functional groups likely to coordinate with Fe (III), such as carboxylic and aromatic domains.

Thus, the loss of spectral resolution with increasing Fe/C ratio in our HA samples results from two factors. The first is direct paramagnetic broadening; the second is structural rearrangement within the humic matrix, which brings protonated groups closer to the Fe (III) ions. These interactions result in both chemical and physical proximity effects that ultimately shape the observed NMR profiles.

### 3.2 Impact of Fe (III) Complexation on Humic Acid Structure: Insights From *T*_*CH*_ Dynamics and Exponential Decay Analysis

**FIGURE 2:**
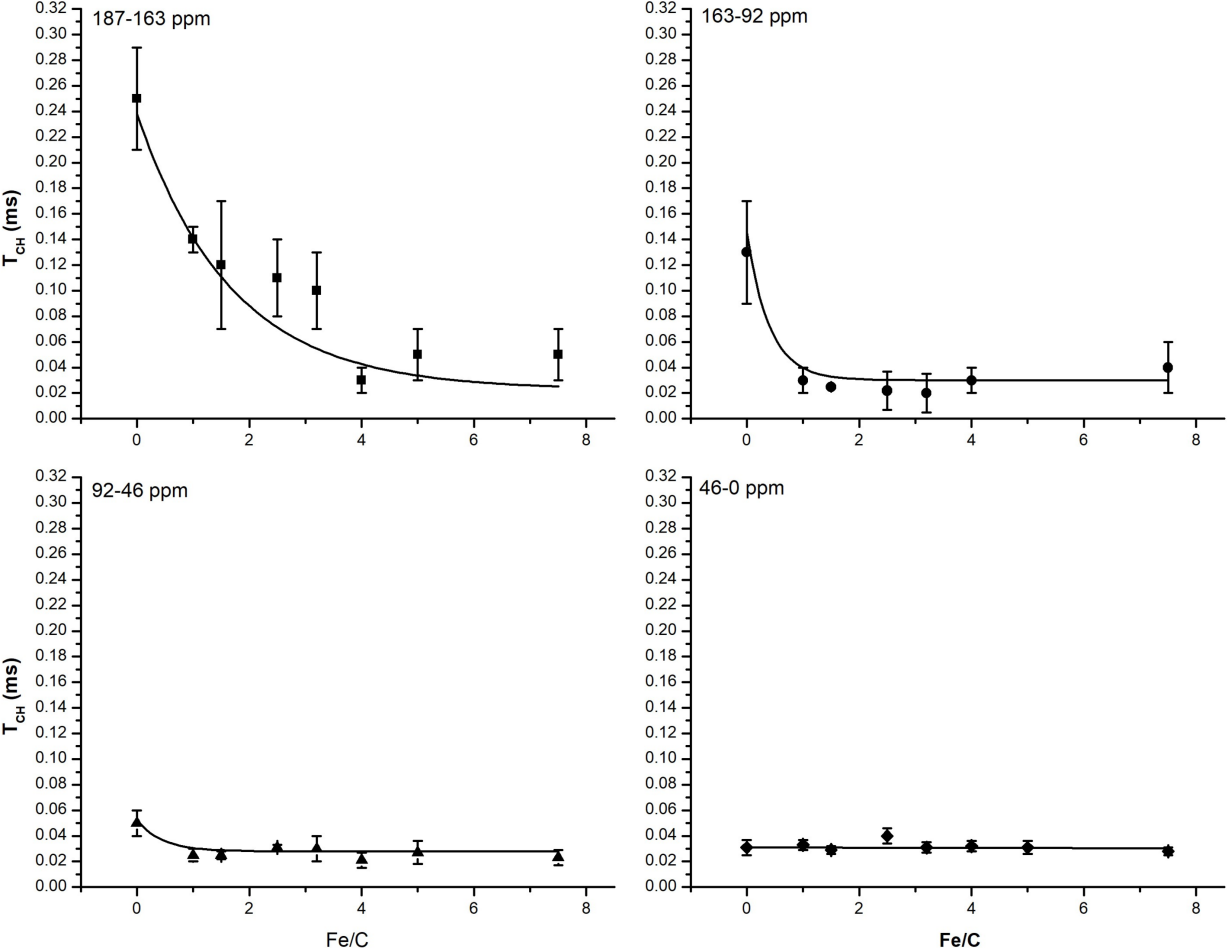
Exponential variation of the *T*_*CH*_ values of the different chemical shift intervals with the Fe/C ratio. The reader is addressed to the main text for the discussion on this exponential decay.

The interaction between HAs and metal ions represents a fundamental process governing organic matter behavior in environmental systems. To elucidate the structural consequences of Fe (III) complexation, we analyzed the cross‐polarization time constant (*T*_*CH*_) as a function of Fe/C ratio using ^13^C CPMAS NMR spectroscopy (Figure 2). This approach reveals a progressive decrease in *T*_*CH*_ across all spectral regions, highlighting the selective nature of Fe (III)–HA interactions.

**FIGURE 3:**
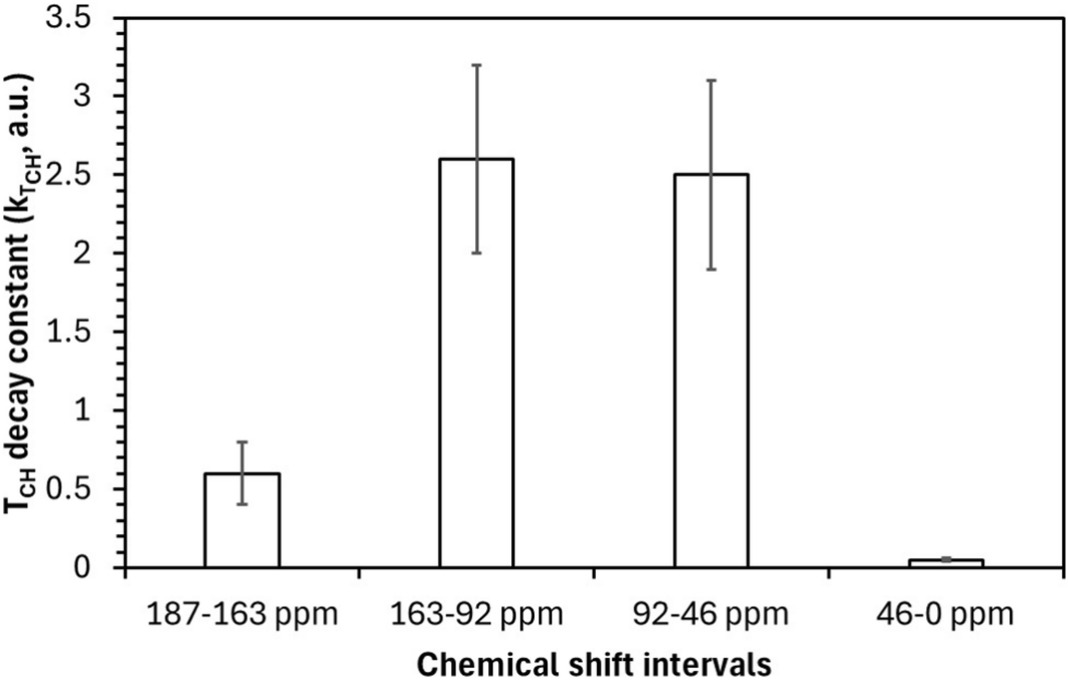
Decay constant (*k*_*TCH*_) for the *T*_*CH*_ values obtained by applying Equation (1) to the VCT experiments.

The most pronounced response occurs in the carboxyl‐rich region (187–163 ppm), where *T*_*CH*_ values drop sharply at low Fe/C ratios before reaching a plateau (Figure 2). Exponential fitting (Figure 2) yields the highest decay constant (*k*_*TCH*_ = 3.5, Figure 3) for this region, underscoring the role of carboxylates as primary Fe (III) binding sites. This behavior likely arises from two complementary effects: (i) local compaction around Fe–carboxylate complexes enhances proton–carbon proximity and (ii) metal–ligand bonding introduces structural rigidity—both of which improve cross‐polarization efficiency. The plateau reflects the saturation of high‐affinity binding sites.

The aromatic region (163–92 ppm) also shows a substantial *T*_*CH*_ reduction, though less abrupt than for carboxylates. The intermediate decay constant (*k*_*TCH*_ = 2.5, Figure 3) suggests secondary Fe (III) coordination via cation–π interactions or phenolic chelation. The steady decline depicted in Figure 2 implies a progressive reorganization of aromatic domains to optimize proton–carbon contacts, albeit less effectively than in carboxyl‐rich regions.

For oxygenated aliphatics (92–46 ppm), *T*_*CH*_ values decrease more gradually, consistent with a modest decay constant (*k*_*TCH*_ = 1.5, Figure 3). This likely reflects indirect effects, such as hydrogen‐bonding network rearrangements, rather than direct Fe (III) coordination. In contrast, the aliphatic region (46–0 ppm) exhibits nearly constant *T*_*CH*_ values across the Fe/C range (Figure 2), supported by a minimal decay constant (*k*_*TCH*_ ≈ 0.05), indicating structural isolation from metal‐binding domains.

The exponential model reported in Equation (2), used to quantify the decay behavior (Figure 3), offers crucial insight into the nonlinear structural dynamics induced by Fe (III) binding. The hierarchy of decay constants (3.5 > 2.5 > 1.5 > 0.05) mirrors both the observed spectral trends and the expected binding affinities of HA functional groups. Most structural changes occur at low Fe/C ratios, with diminishing effects at higher concentrations, reflecting a saturation‐like behavior.

Together, Figures 2 and 3 support a model in which Fe (III) acts as a molecular organizer, establishing localized order through carboxylate coordination that propagates into adjacent aromatic systems. These findings have significant environmental implications, potentially explaining organic matter stabilization in iron‐rich soils [45, 46, 47]. The demonstrated sensitivity of *T*_*CH*_ to subtle structural reorganizations, particularly when combined with decay modeling, highlights the power of this NMR approach for probing complex organic systems. As with all NMR‐based studies involving paramagnetic ions, it is important to consider possible signal distortions or line broadening effects, though these were minimized through experimental design and consistent signal processing.

### 3.3 Probing Relaxation Dynamics: *T*_₁ *ρ*_(*H*) as a Complementary Indicator of Fe (III)–Induced Structuring

**FIGURE 4:**
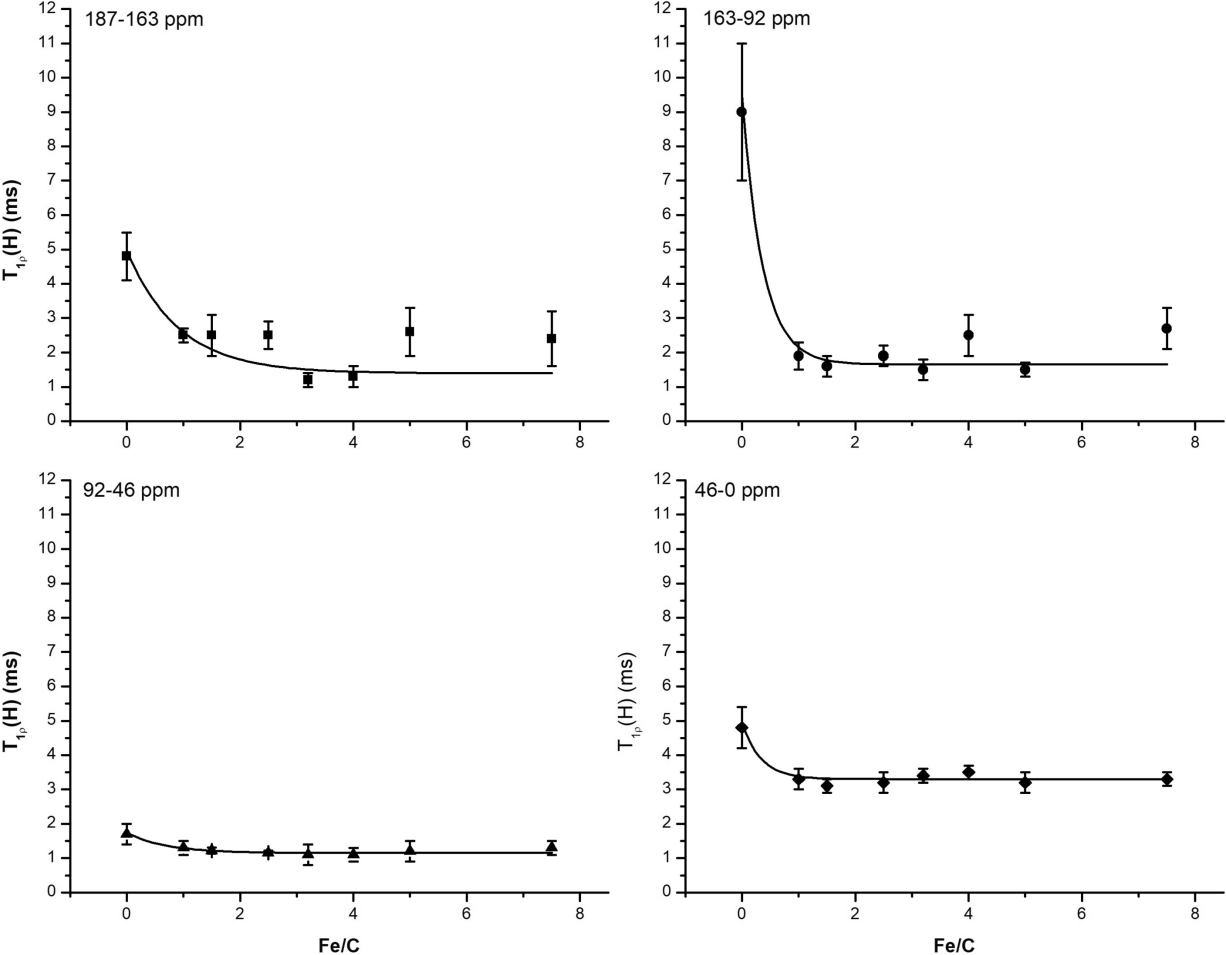
Exponential variation of the *T*_1*ρ*_(*H*) values of the different chemical shift intervals with the Fe/C ratio. The reader is referred to the main text for the discussion on this exponential decay.

To complement the insights obtained from *T*_*CH*_ behavior, we examined the proton spin–lattice relaxation times in the rotating frame (*T*_₁*ρ*_[*H*]) across the same ^13^C CPMAS NMR spectral regions (Figure 4). *T*_1*ρ*_(*H*) is particularly sensitive to local mobility and proton–proton dipolar interactions, offering a dynamic probe of structural reorganization within humic matter upon Fe (III) complexation [43]. As with *T*_*CH*_, the *T*_1*ρ*_(*H*) values exhibit clear Fe/C dependence, with pronounced relaxation changes localized to specific functional domains.

**FIGURE 5:**
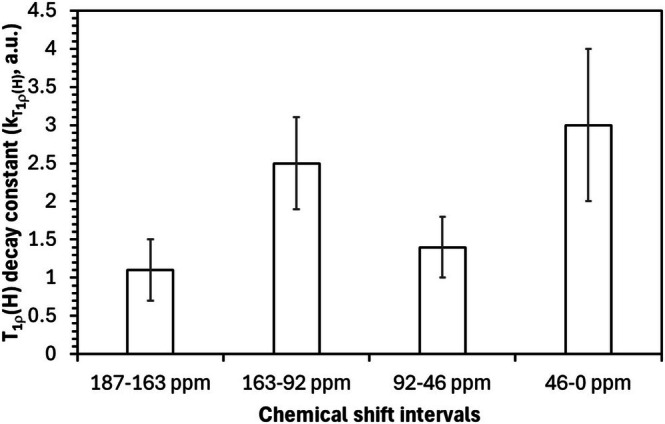
Decay constant (*k*_*T*₁ *ρ* [*H*]_) for the *T*_1*ρ*_(*H*) values obtained by applying Equation (1) to the VCT experiments.

In the carboxyl‐rich region (187–163 ppm), *T*_1*ρ*_(*H*) values decrease rapidly at low Fe/C ratios and stabilize thereafter, consistent with rigidification induced by Fe–carboxylate bonding. This behavior is captured quantitatively by the exponential decay constant *k*_*T*₁*ρ*(*H*)_ = 1.1 (Figure 5), reflecting reduced segmental motion and increased dipolar coupling in these highly coordinated zones. The aromatic region (163–92 ppm) shows the largest decay constant (*k*_*T*₁*ρ*[*H*]_ = 2.5), indicating that once Fe (III) saturates the carboxyl sites, structural stiffening extends into neighboring aromatic domains, possibly through *π*‐stacking realignment or phenolic chelation.

The oxygenated aliphatic region (92–46 ppm) displays a more gradual reduction in *T*_1*ρ*_(*H*), with an intermediate decay constant (*k*_*T*₁*ρ*[*H*]_ = 1.3), suggesting subtler mobility constraints. These may arise from long‐range effects, such as hydrophilic clustering or altered hydration patterns following metal‐induced compaction. Finally, in the aliphatic region (46–0 ppm), *T*_1*ρ*_(*H*) remains largely unchanged as the Fe/C ratio increases. Although the decay constant (*k*_*T*₁*ρ*[*H*]_ = 3.0) is the highest observed, the limited variation in the absolute *T*_1*ρ*_(*H*) values indicates persistent dynamic freedom and minimal interaction with Fe (III) domains.

Although we interpret the decrease in *T*_1*ρ*_(*H*) as indicative of increased molecular rigidity, especially in the carboxyl and aromatic regions, we recognize that paramagnetic relaxation induced by Fe (III) likely constitutes the dominant relaxation mechanism. This effect, arising from enhanced dipolar interactions and electron–nucleus coupling, becomes increasingly significant at higher Fe/C ratios and must be considered alongside structural stiffening in interpreting the relaxation behavior.

The exponential model represented by Equation (2) applied to *T*_1*ρ*_(*H*) data offers valuable quantitative insight into the dynamic landscape of HA during complexation. Interestingly, although the decay constant hierarchy for *T*_1*ρ*_(*H*) (3.0 > 2.5 > 1.3 > 1.1) differs slightly from that of *T*_*CH*_, it still reinforces the dual nature of Fe (III) interactions. Fe (III) simultaneously modifies molecular rigidity (detected by *T*_₁*ρ*_[*H*]) and spatial proximity (detected by *T*_*CH*_). Together, the two parameters reveal that Fe (III) serves not only as a structural binder but also as a regulator of local molecular dynamics.

These findings further support the model of Fe (III) as a molecular organizer within humic matter, with effects that cascade beyond direct coordination sites into the surrounding matrix. The combined use of *T*_*CH*_ and *T*_1*ρ*_(*H*) parameters thus provides a more nuanced understanding of the supramolecular adjustments that humic substances undergo in metal‐rich environments.

## 4 Conclusions

This study reveals that Fe (III) complexation significantly modulates the conformational structure and molecular dynamics of soil‐derived HA, as revealed by ^13^C CPMAS NMR spectroscopy. The progressive degradation of spectral quality and the region‐specific decline in cross‐polarization time constants (*T*_*CH*_) and proton rotating‐frame relaxation times (*T*_₁*ρ*_[*H*]) reflect both direct paramagnetic effects and deeper supramolecular reorganization. The sharp response of carboxyl and aromatic regions to Fe (III) addition underscores their central role in metal binding and structure stabilization, whereas the relatively inert behavior of aliphatic domains confirms their limited involvement in coordination processes.

Quantitative modeling using exponential decay functions provided a mechanistic understanding of how Fe (III) imposes spatial compaction and dynamic rigidity, effectively acting as a molecular cross‐linker within the humic matrix.

**FIGURE 6:**
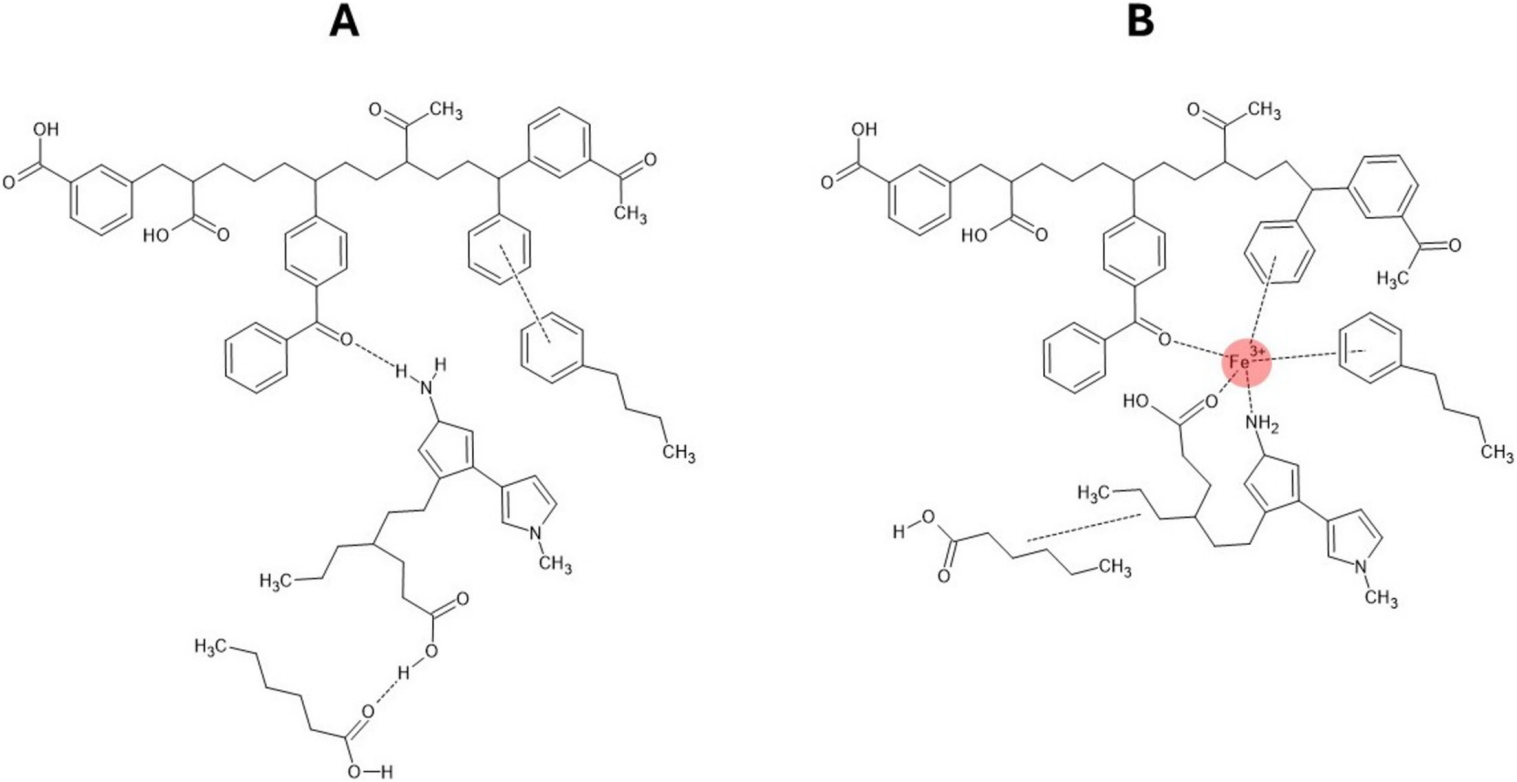
Schematic representation of humic acid (HA) supramolecular organization before (A) and after (B) complexation with Fe (III). In the native state (A), HA exhibits a disordered, loosely associated architecture. Upon Fe (III) coordination (B), carboxylic and aromatic moieties form metal bridges, promoting local compaction and reduced segmental mobility.

Figure 6 schematically illustrates the supramolecular reorganization of HA upon Fe (III) complexation. In the native state (Figure 6A), HA adopts a flexible assembly stabilized by weak interactions such as hydrogen bonding and π–π stacking. Upon coordination with Fe (III) (Figure 6B), local compaction occurs through carboxylate and aromatic interactions, resulting in increased molecular rigidity and spatial ordering of the HA matrix.

The dual use of *T*_*CH*_ and *T*_1__*ρ*_(*H*) as independent yet complementary probes offers a powerful framework for dissecting the interplay between chemical binding and physical structuring in complex natural organic matter. This approach not only enhances our molecular‐level understanding of organo–mineral interactions but also has implications for predicting the fate and stability of organic matter in iron‐rich environments, such as tropical soils and redox‐active sediment systems.

We acknowledge that the spectral aggregation strategy adopted in this study—although effective for capturing average dynamic trends—does not resolve the full heterogeneity of carbon environments in HAs. As demonstrated by Mao et al. [48], advanced spectral editing techniques can provide finer discrimination among overlapping moieties. Although our goal was to monitor bulk supramolecular dynamics rather than to quantify specific functional groups, future work could benefit from integrating variable contact time experiments with spectral editing protocols to better link molecular motion with specific structural features.

Future work should explore how environmental variables—particularly pH, competing metal ions, and redox state—further influence these interactions. Expanding this methodology to a broader range of natural organic matrices could yield valuable insights into the biogeochemical roles of metal–humic associations in terrestrial and aquatic ecosystems.

## Supporting information


**Data S1** Supporting Information

## Data Availability

The data that support the findings of this study are available from the corresponding author upon reasonable request.
