# Supplementary material for: Paramagnetic Probing of Humic Acid Supramolecular Structure: Iron (III)–Induced Rearrangements Revealed by Solid‐State 13C NMR Spectroscopy
Source: Magn Reson Chem. 2025 Jul 10;63(10):768–76. doi: 10.1002/mrc.70011 (PMC12401672; doi:10.1002/mrc.70011)
Supplement: Supplementary file 1 — Data S1 Supporting Information [file MRC-63-768-s001.docx]

# **SUPPLEMENTARY INFORMATION**

# **PARAMAGNETIC PROBING OF HUMIC ACID SUPRAMOLECULAR STRUCTURE: IRON(III)-INDUCED REARRANGEMENTS REVEALED BY SOLID-STATE ^13^C NMR SPECTROSCOPY**

Pellegrino Conte and Calogero Librici

Department of Agricultural, Food and Forest Sciences, University of Palermo, v.le delle Scienze Building 4, 90128 Palermo – Italy

Corresponding author’s e-mail: [pellegrino.conte@unipa.it](mailto:pellegrino.conte@unipa.it)

# **INTRODUCTION**

The following supplementary methods are provided for reproducibility purposes and include detailed descriptions of extraction, characterization, and NMR acquisition protocols.

# **MATERIALS AND METHODS**

# **Extraction and Elemental Characterization of Humic Acid**

After air drying and 2 mm sieving, 200 g of soil were shaken overnight in 500 mL of a 0.5 M NaOH/0.1 M Na_2_P_2_O_7_ solution under N_2_ atmosphere. The mixture was centrifuged and the supernatant filtered through glass wool. The supernatant was first acidified to pH 1.0 with concentrated HCl to precipitate HA, then centrifuged at 7000 rpm to separate the humic acid. This treatment was repeated trice. The HA was, then, shaken for 24 h in a 0.5% HF/0.5% HCl solution in order to remove the co-extracted silicon-containing inorganic components. HA was dialyzed against deionized water till chloride-free and finally freeze-dried. Ash content was measured by heating about 50 mg of HA at 750 °C for 8 h. It was <3%. Elemental composition was measured trice with an elemental analyzer EA 1108 Elemental Analyzer by Fisons’ Instruments (Termoquest, Italy). The averaged carbon, hydrogen and nitrogen contents resulted 56.8 ± 0.3 %, 4.5 ± 0.2 %, and 5.2 ± 0.3 %, respectively.

# **Measurement of the HA acidity and preparation of the Fe-HA complexes.**

An automatic titrator (VIT90 Video Titrator Radiometer, Copenhagen) was used to measure carboxylic and phenol acidity of the HA. Acidity due to carboxyl groups was obtained by titrating a 0.6 mg ml^-1^ HA solution to pH 7.0 with 0.1 M NaOH under a N_2_ stream and stirring. The phenol acidity was measured by titrating the same HA solution up to pH 9.0^1^. According to literature^1^, the total acidity was considered as the sum of the phenol and the carboxyl acidities. Carboxyl, phenol and total acidities were 2.9 ± 0.1, 5.6 ± 0.2 and 8.5 ± 0.3 mmol g^-1^, respectively. All the acidities were obtained by averaging the results of three different measurements. Nine HA solutions titrated at pH 9.0 were kept at this constant pH value and under N_2_ stream while they were added drop-by-drop with a solution of FeCl_3_ (0.1 M) purchased by Sigma^®^ (Milan, Italy). The amount of FeCl_3_ solution to add to each HA solution was calculated in order to correspond to 20, 30, 50, 65, 80, 100 and 150% of the total HA acidity, respectively. No precipitation was clearly visible during the preparation of the complexes. After preparation, the seven Fe-HA solutions were freeze-dried. An aliquot of each solid Fe-humate complex was boiled in a nitric/perchloric acid solution until complete mineralization of the organic matter and dissolution of the inorganic components were obtained. Each solution was transferred to a 20 mL volumetric flask and each sample analysed by atomic adsorption spectrometry (AAS) in order to measure the real content of added Fe. A Perkin–Elmer Analyst 700 instrument (Perkin Elmer, Italy) was used for the AAS measurements. The Fe content was used to retrieve the Fe/C ratio for the samples discussed in the paper.

# **References**

1. Stevenson, F. J. *Humus Chemistry: Genesis, Composition, Reactions*. (Wiley, NY (USA), 1994).

2. Brown, D. E. Fully Automated Baseline Correction of 1D and 2D NMR Spectra Using Bernstein Polynomials. *J. Magn. Reson. Ser. A* **114**, 268–270 (1995).
